# Diffusion phase-imaging in anisotropic media using non-linear gradients for diffusion encoding

**DOI:** 10.1371/journal.pone.0281332

**Published:** 2023-03-30

**Authors:** Pamela Wochner, Torben Schneider, Jason Stockmann, Jack Lee, Ralph Sinkus

**Affiliations:** 1 School of Biomedical Engineering & Imaging Sciences, King’s College London, London, United Kingdom; 2 Philips Healthcare, Guildford, United Kingdom; 3 Athinoula A. Martinos Center for Biomedical Imaging, Harvard Medical School, Massachusetts General Hospital, Charlestown, Massachusetts, United States of America; 4 CRB3, URM773, Inserm, Université Paris Diderot, Sorbonne Paris Cité, Clichy, France; University of North Carolina at Chapel Hill, UNITED STATES

## Abstract

Diffusion MRI classically uses gradient fields that vary linearly in space to encode the diffusion of water molecules in the signal magnitude by tempering its intensity. In spin ensembles, a presumably equal number of particles move in positive and negative direction, resulting in approximately zero change in net phase. Hence, in classical diffusion weighted MRI with a linear gradient field, the phase does not carry any information as the incoherent motion of the spins only impacts the magnitude of the signal. Conversely, when the linear gradient field is replaced with one that varies quadratically over space, the diffusion of water molecules in anisotropic media does give rise to a change in net phase and preserves large portion of the signal around the saddle point of the gradient field. In this work, the phase evolution of anisotropic fibre phantoms in the presence of quadratic gradient fields was studied in Monte Carlo simulations and diffusion MRI experiments. The simulations confirm the dependence of the phase change on the degree of anisotropy of the media and the diffusion weighting, as predicted by the derived analytic model. First MR experiments show a phase change depending on the diffusion time in an anisotropic synthetic fibre phantom, and approximately zero phase change for the experiment repeated in an isotropic agar phantom. As predicted by the analytic model, an increase of the diffusion time by approximately a factor of two leads to an increase of approximately a factor of two in the signal phase.

## Introduction

Diffusion magnetic resonance imaging (dMRI) is a non-invasive imaging modality that exploits the self-diffusion of water molecules to generate image contrast. It is based on Brownian motion, the thermally induced random motion of molecules in liquid or gas. In tissue, the moving molecules encounter and interact with obstacles such as tissue boundaries and cell membranes, which hinder the diffusion process. It is exactly these impediments that give rise to the image contrast in dMRI. Sensitising the MR signal to diffusion allows the molecules to probe their environment while they diffuse, thus providing valuable information about the underlying tissue microstructure. Usually, this is achieved by applying strong diffusion gradients that vary linearly over space. The diffusion is effectively encoded in the attenuation of signal magnitude. dMRI offers unique insight into tissue structure not available to other imaging modalities, making it a popular choice for both clinical and research applications. It is routinely used for the diagnosis and management of ischemic stroke [1, 2]. In diffusion tensor imaging (DTI), images for six or more different diffusion gradient directions are acquired. The major eigenvector of the diffusion tensor gives the principal direction of diffusion in anisotropic media. In tractography, this allows the reconstruction of the trajectories of white matter (WM) bundles and can provide insight into brain connectivity, or to assess pathologies [3, 4].

All of these methods are based on the attenuation of signal magnitude with strong linear gradients. Especially for strong diffusion weightings with high gradient strengths,this can lead to limited sensitivity to mictrostructure. Recent studies have shown, however, that strong diffusion weightings are key to overcome the resolution limit in dMRI and to assess small changes in tissue microstructure, e.g. axon size or cancer cell radii [5, 6]. In this paper, we propose a completely novel approach that uses a non-linear gradient field. In doing so, the diffusion weighting is encoded in the phase of the MR signal and the signal magnitude is largely preserved potentially allowing much stronger diffusion weighting than possible with linear gradients.

Non-linear gradient fields are usually treated as a nuisance in dMRI [7]. The very few constructive applications in the field of MRI have been reported for shimming [8] and spatial encoding [9, 10]. Replacing the typical linear diffusion gradient with a gradient field that changes quadratically in space, diffusion can be encoded in the phase of the MR signal while preserving the signal magnitude around the centre of the gradient field. Away from the saddle point the linear components of the gradient field dephase the signal. We study the phase evolution of a spin ensemble under the influence of such a quadratic gradient field in theory, in Monte Carlo simulations and in dMRI experiments. Due to Maxwell’s equations $\nabla^{2}\vec{B}=0$, a net phase can only be observed for spatially anisotropic diffusion. The here derived analytic model shows a phase change for anisotropic diffusion depending on properties of the underlying media and on the diffusion weighting. If diffusion is isotropic, no change in net phase is expected. This is supported by the findings of simulation and dMRI experiments on anisotropic and isotropic phantoms.

The rest of this paper is structured as follows: The section Analytic Model introduces the theoretical framework required to derive an analytic model which allows first estimates of the change in net phase due to diffusion in an idealised setting. Then the Monte Carlo simulation framework and validation simulations to establish its validity are described. Moreover, simulations in numerical fibre phantoms are described. In Section DMRI Experiments, the experimental set-up, in particular the gradient coil that generates the non-linear gradient, is described before discussing the synthetic fibre phantom and the dMRI experiments. Measurements acquired in an anisotropic synthetic fibre phantom are compared to measurements in isotropic agar gel.

## Analytic model

**Fig 1:**
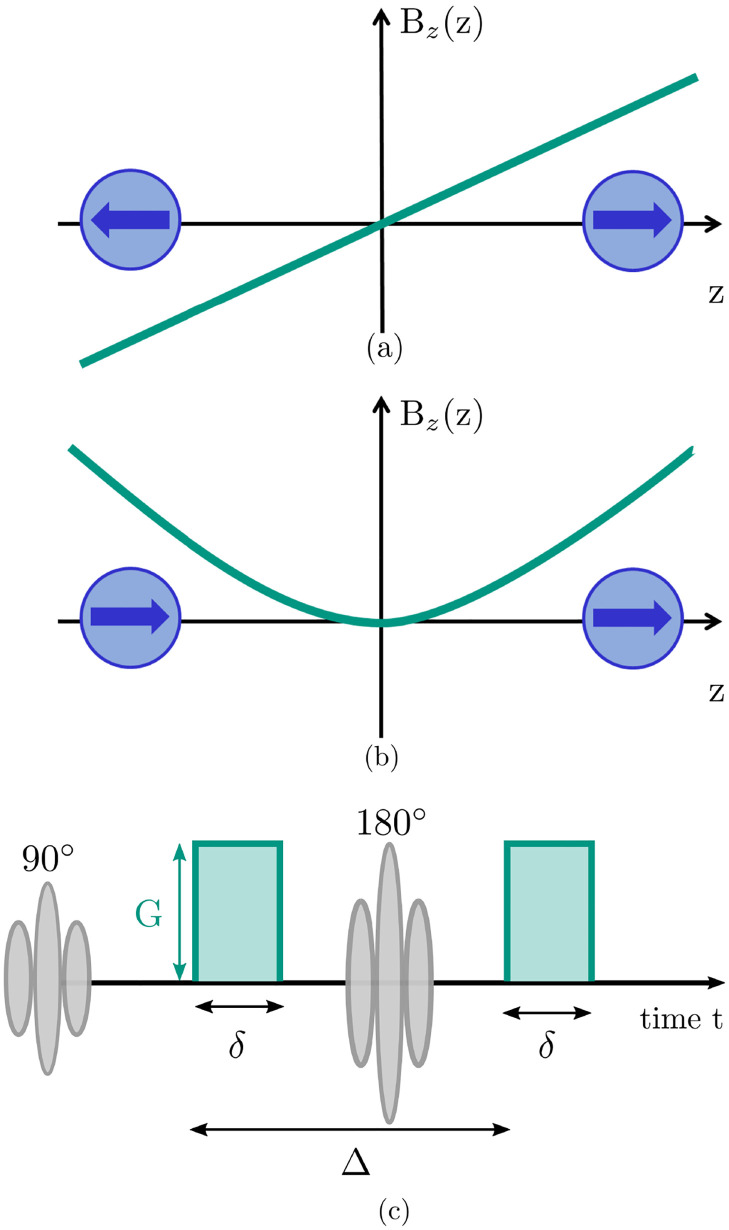
The schematic of one-dimensional gradient fields, with (a) linear and (b) quadratic spatial variation. The green line represents the gradient. In (c), the RF pulses (grey) and the on/off switching of the gradients is shown. The first diffusion gradient pulse of duration *δ* is followed by a second identical pulse. The 180° pulse is effectively inverting the polarity of the second gradient pulse. Δ is the separation time between the two gradient pulses.

The magnetic field $\vec{B}$ generated by the gradient coil is, like all magnetic fields, a vector field $\vec{B}=(B_{x},B_{y},B_{z})$, where the subscript denotes the axis along which the component is aligned. In MRI, for simplification only the *B*_*z*_ component is typically considered. Fig 1(a) shows a typically used linear gradient field *B*_*z*_ = *G*_1_*z*, here applied along the z-direction, and (b) a hypothetical quadratic gradient field *B*_*z*_ = *G*_2_*z*^2^. *G*_1_ describes the magnetic field gradient in T m^-1^ for the linear field, and *G*_2_ is the curvature in Tm^-2^ for the quadratic field, *z* describes the displacement in z-direction. To illustrate the basic idea of this new method, both gradient fields are, for the moment, assumed to be one-dimensional. That means the magnetic field only spatially varies along the z-direction. It should be highlighted that in reality this is not possible for the quadratic field, which will be discussed in more detail later.

In a typical pulsed field gradient spin echo (PFGSE) experiment [11], a strong linear diffusion gradient is applied, which dephases the spins almost instantaneously. Another identical gradient pulse is used to refocus static spins. Spins that have moved do not experience the same magnetic environment as before and do not re-phase. In order to investigate the effect of this on the MR signal, first the phase *φ*_*s*_ an individual spin accumulates while diffusing under a gradient field for the time *T* is considered [12]. *φ*_*S*1_ gives the phase accumulation for a linear gradient field

$$\begin{array}{c} \varphi_{S1}\left(t\right)=\gamma \int_{0}^{T}d\tau \;\;G_{1}\left(\tau \right)z\left(\tau \right)\; \end{array}$$ (1)

with *γ* being the gyromagnetic ratio. For a quadratic gradient field, this has to be modified accordingly:

$$\begin{array}{c} \varphi_{S2}\left(t\right)=\gamma \int_{0}^{T}d\tau \;\;G_{2}\left(\tau \right)z^{2}\left(\tau \right)\;. \end{array}$$ (2)

Fig 1 illustrates the principal idea of the novel approach to dMRI proposed here: For free diffusion, when a spin ensemble starts to diffuse from the origin, a presumably equal number of spins move in positive and negative direction. As shown in Fig 1(a), for a linear gradient field positive and negative phase shifts cancel each other out, while the phase shifts are coherent in case of the quadratic gradient field shown in Fig 1(b).

This can be also shown analytically. Eq 1 allows the calculation of the phase accumulation of an individual spin. For the MR signal, however, many spins have to be considered. Since it is impossible to trace each spin individually, we use the concept of an ensemble. [13] An ensemble is the set of all possible outcomes of a system and the ensemble average, which is analogous to the expected value of the outcomes, is given by

$$\begin{array}{c} E[X(t)]=\int_{-\infty }^{\infty }dx\;x\cdot p_{X(t)}\left(x\right) \end{array}$$ (3)

*p*_*X*(*t*)_ is the probability density function (PDF) of the stochastic process *X*(*t*) and x is the state of the system. In our case, the stochastic process is one-dimensional free diffusion in homogeneous media and the probability of a particle to be at a certain time t and a certain location z, where the location of a particle is the state of the system, is given by

$$\begin{array}{c} \begin{array}{cc} p(z,t)= & \frac{1}{\sqrt{4\pi Dt}}\text{exp}(-\frac{z^{2}}{4Dt})\;. \end{array} \end{array}$$ (4)

assuming that the particle is located at *z* = 0 for *t* = 0. Based on a linear gradient field, an analytic model for the net phase *φ*_1_ of a spin ensemble is derived by combining Eq 3 with Eq 1, where the possible spin displacements represent the stochastic process with the PDF given in Eq 4:

$$\begin{array}{c} \begin{array}{cc} \varphi_{1} & =\gamma \int_{0}^{T}dt\int_{-\frac{\Delta z}{2}}^{+\frac{\Delta z}{2}}dz\;\;G_{1}\frac{z}{\sqrt{4\pi Dt}}\text{exp}(-\frac{z^{2}}{4Dt})\\ & =0. \end{array} \end{array}$$ (5)

Δ*z* is the size of the imaging voxel in z-direction.

The analytic model consists of two integrals: one over space and one over the time of the diffusion process. The solution of the temporal integral depends on the on and off switching of the diffusion gradient pulses, and is here based on a typical PFGSE sequence (see Fig 1(c)). Since the spatial integral is an integral of an anti-symmetric function with symmetric integration borders, it is zero and consequently also the whole expression. Instead, the spins dephase with the degree of dephasing depending on the displacement from their original position. This ultimately results in an attenuation of the signal magnitude due to diffusion given by

$$\begin{array}{c} \begin{array}{c} S(b)=S_{0}\text{exp}\left(-bD\right) \end{array} \end{array}$$ (6)

for a PFGSE diffusion experiment, where *b* is given by $\gamma^{2}G_{1}^{2}\delta^{2}(\Delta -\delta /3)$. The b-value summarises gradient effects and gives a measure for the amount of diffusion-weighting [14]. *S*_0_ is the signal magnitude without diffusion weighting, *D* is the diffusion coefficient, *δ* the duration of the gradient pulse and Δ is the separation time between the start of the first and the second diffusion gradient pulse (see Fig 1(c)). The net phase remains unchanged (*φ*_1_ = 0) [12].

The situation changes, however, when a quadratic diffusion gradient is applied (Fig 1(b)). In this case, spins experience a phase shift of the same polarity independent of the direction of their motion along z. Ultimately, the spin ensemble accumulates a non-zero net phase due to diffusion that contains information about the probed microstructure.

Analogous to Eq 5, an analytic model for the net phase *φ*_2_ can be derived based on a 1D quadratic gradient field by combining Eq 2 with Eq 3 and the PDF in Eq 4:

$$\begin{array}{c} \begin{array}{cc} \varphi_{2} & =\gamma \int_{0}^{T}dt\int_{-\frac{\Delta z}{2}}^{+\frac{\Delta z}{2}}dz\;G_{2}z^{2}\frac{1}{\sqrt{4\pi Dt}}\text{exp}(-\frac{z^{2}}{4Dt})\\ & =\gamma G_{2}\int_{0}^{T}dt\;2Dt\;. \end{array} \end{array}$$ (7)

For details on the solution of the spatial integral, see S1 File. The solution of the temporal integral depends on the on and off switching of the gradients as shown in Fig 1(c). This yields

$$\begin{array}{c} \begin{array}{cc} \varphi_{2} & =2\gamma G_{2}D(\int_{0}^{\delta }dt\;t-\int_{\Delta }^{\delta +\Delta }dt\;t)\\ & =-2\gamma G_{2}D\delta \Delta \;. \end{array} \end{array}$$ (8)

The minus between the two integrals is due to the effect of the refocusing pulse. The obtained model for one-dimensional diffusion predicts a phase change that depends on the properties of the underlying medium and the diffusion weighting.

In order to extend the analytic model to three-dimensional diffusion, the shape of the quadratic gradient has to be considered in three dimensions as well. Gradient fields, like all magnetic fields, are governed by Maxwell’s equations which in free space simplify to the Laplace equation $\nabla^{2}\vec{B}=0$ [15]. For the design of the magnetic field we want to generate with the gradient coil we focus on *B*_*z*_. The possible shapes of *B*_*z*_ are hence given by the solutions to ∇^2^*B*_*z*_ = 0. As described in detail by Chmurny and Hoult [16], this equation has many solutions of different orders. For the purpose of the presented method, a second order magnetic field with a dominant component along z was chosen, which can be described by

$$\begin{array}{c} B_{z}\left(x,y,z\right)=G_{2}(z^{2}-\frac{1}{2}\left(x^{2}+y^{2}\right)) \end{array}$$ (9)

where *G*_2_ is the curvature of the field in Tm^-2^. As mentioned earlier, the subscript only indicates the field alignment with the z-axis; the field has spatial variations with respect to all three directions and a saddle point at the origin. In contrast, linear gradients that solve the Laplace equation vary only in one direction. Non-linear gradient fields of different orders and shapes are used in the field of *B*_0_ shimming and a gradient field which has a shape described by Eq 3 is commonly abbreviated with the shorthand Z2 [16].

By extending the analytic model to three-dimensional diffusion based on a Z2 gradient field, and assigning an individual diffusion coefficient to each direction (*D*_*x*_, *D*_*y*_, *D*_*z*_), we obtain

$$\begin{array}{c} \begin{array}{cc} \varphi_{2,3D} & =-2\gamma G_{2}\delta \Delta (D_{z}-\frac{1}{2}\left(D_{x}+D_{y}\right))\;. \end{array} \end{array}$$ (10)

If cylinder symmetry is assumed, a transversal diffusion coefficient *D*_⊥_ and a longitudinal diffusion cofficient *D*_∥_ can be introduced with *D*_⊥_ = *D*_*x*_ = *D*_*y*_ and *D*_∥_ = *D*_*z*_. Inserted into Eq 10 this yields

$$\begin{array}{c} \begin{array}{cc} \varphi_{2,3D} & =-2\gamma G_{2}\delta \Delta D_{\|}(1-\frac{D_{⊥}}{D_{\|}})\;. \end{array} \end{array}$$ (11)

A few assumptions were made for this derivation:

- All spins start to diffuse at time t = 0 from the origin. Spins that do not start from the origin should have a similar trend.
- Diffusion is locally constant.
- Both gradient pulses have rectangular shape and the same duration and identical gradient magnitude.

For further details on the derivation see S1 File.

The analytic model in Eq 10 suggests that if diffusion is isotropic, i.e. it does not have a preferred direction (*D*_*x*_ = *D*_*y*_ = *D*_*z*_), the analytic phase model yields zero. For anisotropic diffusion, however, a phase shift is expected that can provide information about the underlying anisotropic media.

## Monte Carlo simulations

In the following, a Monte Carlo simulation framework is described to study the behaviour of a spin ensemble diffusing in arbitrary geometries in the presence of a Z2 gradient field. This allows the simulation of the bulk MR signal of structures that are too complex to describe analytically.

First, the trajectories for each of the *N* spins in the ensemble is simulated using existing Monte Carlo open-source software toolkits. The Brownian motion library by Burkardt [17] was used to validate the proposed analytic model against the simulation of free diffusion. Simulations in a cylinder substrate mimicking a synthetic fibre phantom were done with the Camino toolkit [18], which allows the performance of Monte Carlo diffusion simulations in arbitrary geometries represented by polyhedral meshes.

The individual spin trajectories were then used to determine the phase accumulation of each spin, and ultimately the net phase change of the spin ensemble as follows: the total duration of the simulation is divided into *T*_*s*_ time steps of duration *Δt*. The effect that the gradient field has on an individual spin during each time step is computed using Eq 2, which requires the position of the spin dependent on the time variable *t*. For simplification, we assume a spin to move from its position at the start of the time step ${\vec{r}}_{n}$ = (*x*_*n*_, *y*_*n*_, *z*_*n*_) to its new position ${\vec{r}}_{n+1}$ = (*x*_*n*+1_, *y*_*x*+1_, *z*_*n*+1_) in a straight line, i.e. the position of the particle along the z-axis *z*(*t*) during a time step is given by

$$\begin{array}{c} z\left(t\right)=z_{n}+\frac{t}{\Delta t}\left(z_{n+1}-z_{n}\right)\;. \end{array}$$ (12)

The positions along x- and y-axis can be found accordingly. Combined with Eq 2, we obtain the phase *φ*_sim_ a single spin accumulates during one time step:

$$\begin{array}{c} \begin{array}{cc} \varphi_{\text{sim}}= & C\gamma \int_{0}^{\Delta t}dt\;G_{2}({\left(z_{n}+\frac{t}{\Delta t}\left(z_{n+1}-z_{n}\right)\right)}^{2}\\ & -\frac{1}{2}{\left(x_{n}+\frac{t}{\Delta t}\left(x_{n+1}-x_{n}\right)\right)}^{2}-\frac{1}{2}{\left(y_{n}+\frac{t}{\Delta t}\left(y_{n+1}-y_{n}\right)\right)}^{2})\\ = & C\gamma G_{2}\Delta t(z_{n}z_{n+1}+\frac{1}{3}{\left(z_{n+1}-z_{n}\right)}^{2}\\ & -\frac{1}{2}(x_{n}x_{n+1}+\frac{1}{3}{\left(x_{n+1}-x_{n}\right)}^{2})\\ & -\frac{1}{2}(y_{n}y_{n+1}+\frac{1}{3}{\left(y_{n+1}-y_{n}\right)}^{2}))\;. \end{array} \end{array}$$ (13)

Note that the factor $\frac{1}{2}$ originates from the shape of the gradient field (Eq 9). Moreover, *C* is a constant that refers to the polarity of the applied gradient pulse and is +1 for the duration of the first gradient pulse, -1 for the second, and 0 in between. This change in polarity accounts for the effect of the refocusing pulse. The phase contributions for each spin and time step are accumulated and the phase of a particle after all time steps is written to a phase histogram. After all individual phase accumulations have been determined as described, the net phase is found as the mean of the Gaussian distribution fitted to the phase histogram.

### Validation simulations

This set of simulations aims to establish the validity of the proposed analytic model via the simulation framework established above. The following cases were simulated:

(1) isotropic diffusion with varying diffusion weighting (expected to yield no change in net phase) (2) anisotropic diffusion with varying diffusion weighting and constant degree of anisotropy, and (3) anisotropic diffusion with varying degrees of anisotropy and constant diffusion weighting.

#### Methods

The simulation set-up was chosen such that it reflects the assumptions made in the model. The diffusion coefficients are locally constant and all spins were initially positioned at the centre of the voxel. Since the simulated diffusing particles represent water molecules, the values for the gyromagnetic ratio and the diffusion coefficient *D*_∥_ (at 25°C) were based on values found in the literature [19].

All parameters are summarised in Table 1. Each of the simulations A1–3 is performed ten times with different trajectories for each parameter combination.

**Table 1:** The simulation parameters used in the validation simulations.

Parameter	Simulation	Value	Unit
Gyromagnetic ratio *γ*	A1–3	2.6×10^8^	rad s^-1^ T^-1^
Diffusion coefficient *D*_∥_	A1–3	2.3×10^−9^	m^2^ s^-1^
Ratio diffusion coefficients *D*_⊥_/*D*_∥_	A1	1	
	A2	0.5	
	A3	0.1–0.9	
Curvature *G*_2_	A1–3	25	T m^-2^
Gradient pulse duration *δ*	A1–2	10–50	ms
	A3	20	ms
Separation time Δ	A1–2	260—300	ms
	A3	270	ms
Number of particles *N*	A1–3	10^5^	
Time steps *T*_*s*_	A1–3	10^3^	

*Simulation A1*: Isotropic diffusion was chosen (*D*_⊥_ = *D*_∥_) and the diffusion weighting was varied changing the duration of the gradient pulse *δ*. The time Δ_*sep*_ between the end of the first gradient pulse and start of the second was constant, but Δ = Δ_*sep*_+ *δ* varies with *δ*.

*Simulation A2*: For the second experiment, anisotropic diffusion with a constant ratio *D*_⊥_/*D*_∥_ was chosen and the diffusion weighting was varied as described in simulation A1.

*Simulation A3*: The diffusion weighting was kept constant while the degree of anisotropy is changed by varying the ratio *D*_⊥_/*D*_∥_ from 0.1 to 0.9 in steps of 0.1.

#### Results

**Fig 2:**
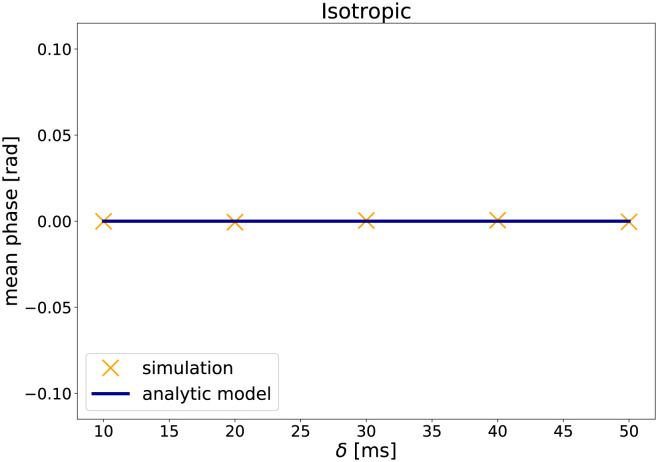
The results of the validation simulation A1: The mean net phase versus the duration of the gradient pulse *δ* for isotropic diffusion.

**Fig 3:**
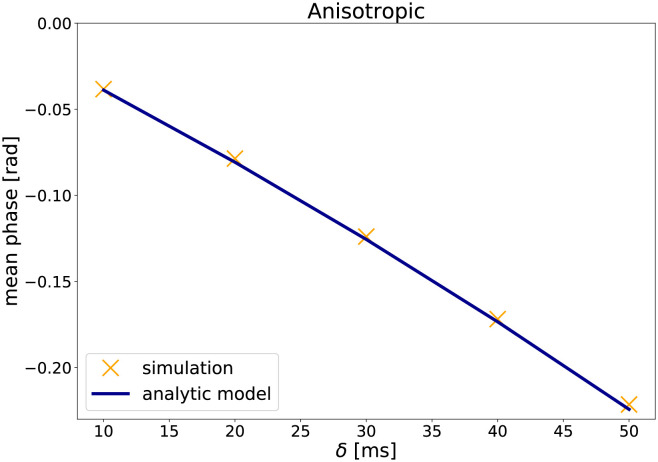
The results of the validation experiment A2: The mean net phase versus the duration of the gradient pulse *δ* for anisotropic diffusion (*D*_⊥_/*D*_∥_ = 0.5).

Fig 2 shows the mean net phase in dependence of the duration of the gradient pulse *δ* for isotropic diffusion (simulation A1), while Fig 3 shows the results for the anisotropic case (simulation A2). The plots also show the net phase as predicted by the analytic model. The phase changes predicted by the analytic model agree with the simulated phase changes, which is also supported by the mean squared error (MSE) between net phase obtained by simulation and predicted by the analytic model (see Table 2).

**Table 2:** The MSE of the net phase between the simulation and the analytic model for the validation simulations.

Simulation	MSE	Unit
A1	3.82×10^−6^	rad^2^
A2	3.47×10^−6^	rad^2^
A3	1.41×10^−6^	rad^2^

As expected from the analytic model, isotropic diffusion results in approximately zero phase change. In contrast, for anisotropic diffusion, an increase of diffusion-weighting, here in form of longer gradient pulses *δ*, leads to an increase in net phase.

**Fig 4:**
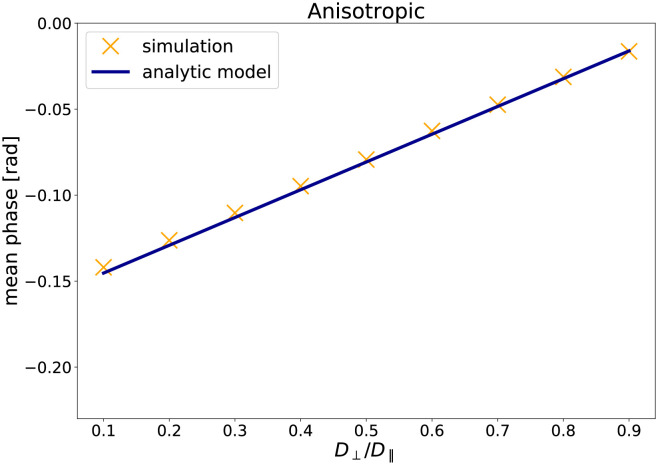
The results of the validation simulation A3: The mean net phase versus *D*_⊥_/*D*_∥_ for 3D diffusion.

Fig 4 shows the mean net phase as a function of the ratio *D*_⊥_/*D*_∥_ and the net phase as predicted by the analytic model (simulation A3). As for the simulations before, the simulated phase accumulations in the simulations agree with the phase change predicted by the analytic model. Moreover, the results show that the net phase increases with increasing anisotropy of the phantom, with a low ratio *D*_⊥_/*D*_∥_ corresponding to high anisotropy and therefore a large phase change.

The validity of the analytic model was established by comparing the results to the phase changes obtained from the validation simulations. In addition, the dependence of the net phase on both the diffusion weighting and on properties of the underlying medium were confirmed. Based on this finding, the simulation can be extended to consider situations that go beyond the model but better reflect realistic scenarios.

### Numerical fibre phantom simulations

In reality, anisotropic phantoms are built by adding barriers to an isotropic substance that is MR visible. The phantom later used for dMRI experiments is based on the work published by Fieremans et al. [20, 21]. They designed a synthetic fibre phantom that allows the quantitative validation of dMRI methods. The properties of the phantom were determined in dMRI experiments and these measurements were then compared to Monte Carlo simulations in a numerical fibre phantom consisting of randomly packed parallel cylinders with Gaussian-distributed diameters. A similar phantom was created by fitting a gamma-distribution such that it approximates the Gaussian-distributed cylinder radii with mean *μ* = 20 μm and standard deviation *σ* = 4.1 μm. The parallel cylinders are infinitely long and aligned with the z-axis. The thus obtained scale parameter k and shape parameter *θ* (see Table 3) were then used to generate a substrate consisting of a fixed number of non-overlapping infinitely long cylinders parallel to the z-axis with random packing geometry. The substrate size was constant. The substrates are periodic along the edges, i.e. overlapping cylinders are simply continued on the opposite site. In doing so, spins that diffuse beyond the substrate boundaries can simply enter the substrate again on the opposite site [22]. Since synthetic fibres are hydrophobic, the spins were distributed randomly across the extracellular space of the substrate. The duration and separation of the gradient pulses were chosen to match the ones in the later described dMRI experiments, where a stimulated echo mode (STEAM) sequence is used to achieve long separation times (more details in section Experiments).

**Table 3:** Physical and scan parameter for simulations on the numerical fibre phantom.

Parameter	Simulation	value	unit
Scale parameter k	B1–2	4.72×10^−6^	
Shape parameter *θ*	B1–2	21.1	
Substrate size (x, y, z)	B1–2	(5.01, 5.01, 5.01) × 10^−4^	m
Number of cylinders	B1	200–500	
	B2	500	
Diffusivity *D*	B1–2	2.3×10^−9^	m^2^ s^-1^
Gradient pulse duration *δ*	B1–2	20	ms
Separation time Δ	B1	270	ms
	B2	270—570	ms
Curvature gradient field *G*_2_	B1–2	25	T m^-2^
b-values	B1	[576 712 1025]	s mm^-2^
b-values	B2	[800 1600]	s mm^-2^
G_1_	B1	0.028–0.037	T m^-1^
	B2	0.007- 0.014	T m^-1^
Number of particles *N*	B1–2	1.5×10^5^	
Number of time steps *T*_*s*_	B1–2	2.0×10^3^	
Spin initialisation	B1–2	extracellular	

The following simulations with the numerical fibre phantom aim to investigate the dependence of the net phase on both physical properties of the phantom and the scan parameters. In order to do so, two cases were considered: (1) the influence of the fibre density on the net phase for constant diffusion weighting, and (2) the influence of the diffusion weighting on the net phase for constant fibre density.

#### Methods

Both cases were simulated with quadratic gradients as well as linear gradients. In simulations, it is possible to obtain, next to the net phase, also the individual phase contributions of all three directions. These can be used to determine the diffusion coefficients *D*_*x*_, *D*_*y*_, *D*_*z*_ (using Eq 8). It has been shown that the motion parallel and transverse to the cylinders is statistically independent. This means that the DT is a diagonal matrix with *D*_*x*_, *D*_*y*_ and *D*_*z*_ being the eigenvalues [23, 24], which can be used to compute the fractional anisotropy (FA), a measure to quantify the degree of anisotropy. Although it is not possible to obtain the directional phase components, and hence the FA, from the later described dMRI experiments, the FA obtained from the simulations is useful to confirm the validity of the simulations, as it provides a way to compare results from simulations with quadratic diffusion gradients to those obtained with linear gradients. The b-values in the simulations with linear gradients were composed such that pulse duration *δ* and separation time Δ agree with those used in the simulations with quadratic gradients.

*Simulation B1*: The number of cylinders is increased in steps of 100 while keeping the substrate size constant. This reduces the space between the cylinders and therefore increases the fibre density. The diffusion coefficients are retrieved from the simulated net phase in each direction and subsequently used to compute the FA. All simulations were performed ten times for each considered parameter configuration with different spin trajectories, and the parameters are listed in Table 3. Synthetic dMRI measurements for the FA with linear gradients were generated with Camino. For each fibre density, three b-values were used.

*Simulation B2*: For a substrate with a constant number of fibres (and hence fibre density), the diffusion weighting was varied by increasing the separation time Δ between two gradient pulses. Besides the simulations with quadratic gradients, simulations with linear gradients were performed with two b-values.

#### Results

**Fig 5:**
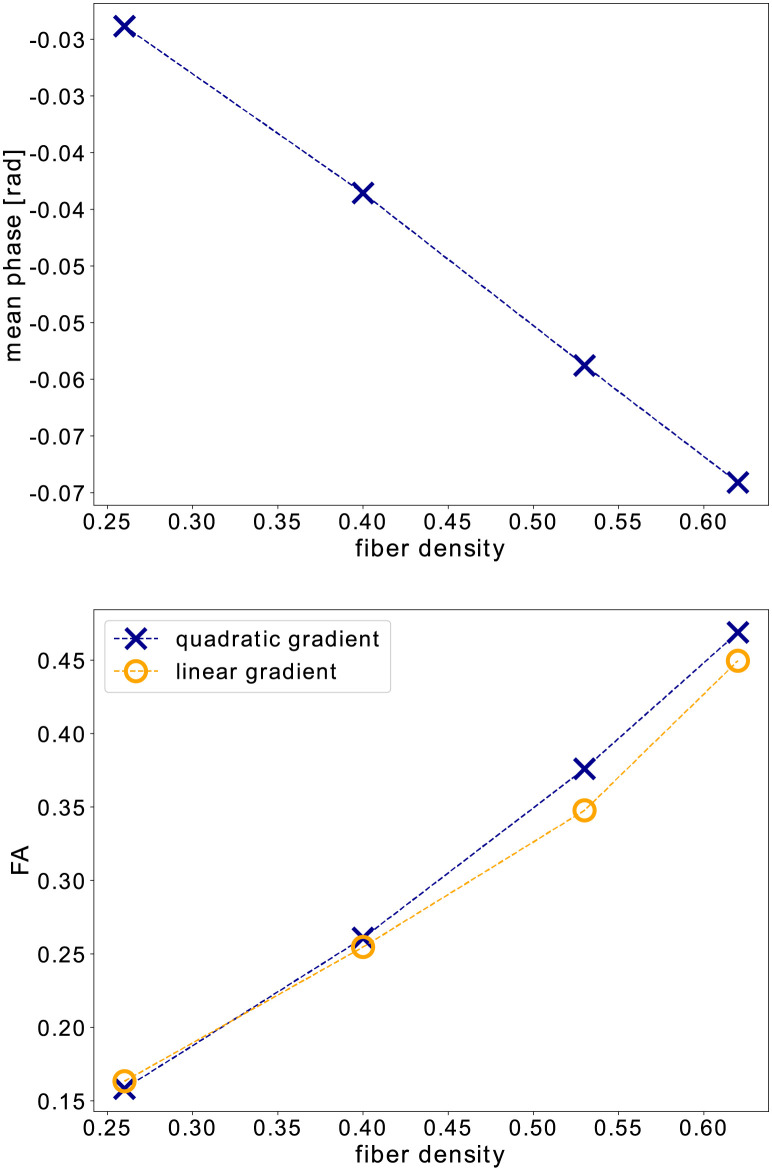
The results of the numerical fibre phantom experiment B1: The mean net phase (top) and FA (bottom) versus the fibre density FD of the phantom.

The results of simulation B1 can be found in Fig 5. The plot at the top shows the mean phase (over all directions) for different fibre densities, while the plot at the bottom shows the FA obtained with both quadratic and linear gradients. The results show that an increasing fibre density in the phantom leads to an increase in net phase for quadratic diffusion gradients. An increase in fibre density reduces the extracellular space and, therefore, limits the diffusion in x- and y-direction. Along the cylinders, diffusion is still free. This is also reflected in the FA values, which increase with the fibre density. FA values obtained with quadratic gradients are comparable to those from simulations with linear gradients, which is confirmed by the MSE in Table 4.

**Table 4:** The MSE between the FA values obtained with quadratic and linear gradients for the numerical fibre phantom.

Simulation	Initial spins	MSE
B1	extracellular	1.3×10^−3^
B2	extracellular	6.58×10^−4^

**Fig 6:**
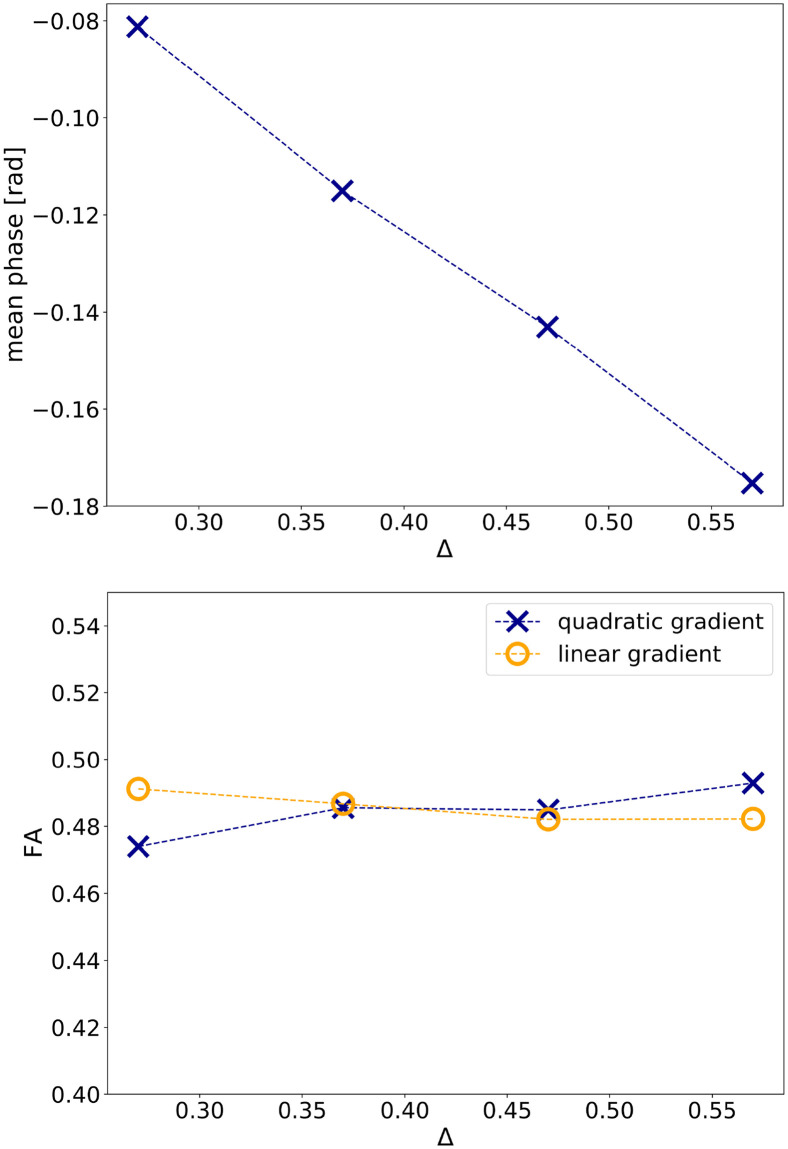
The results of the numerical fibre phantom experiment B2: The mean net phase (top) and FA (bottom) versus the separation time Δ between the two diffusion gradient pulses.

Fig 6 shows the results for simulation B2: the plot at the top shows the mean net phase in dependence of the separation time Δ, and the plot at the bottom shows the FA for quadratic and linear gradients, also in dependence of Δ. For longer Δ and therefore increased diffusion-weighting, a larger change in net phase is observed. The FA seems to tend to increase slightly with Δ for quadratic gradients while the opposite can be observed for linear gradients. However, the changes are relatively small and overall, linear and quadratic gradients yield similar results (see also Table 4).

## DMRI experiments

Finally, our novel approach to dMRI was evaluated in physical MR scans. In order to enable such experiments, a gradient coil capable of generating a Z2 magnetic gradient field, and also a suitable physical anisotropic phantom were designed.

### Experimental set-up

**Fig 7:**
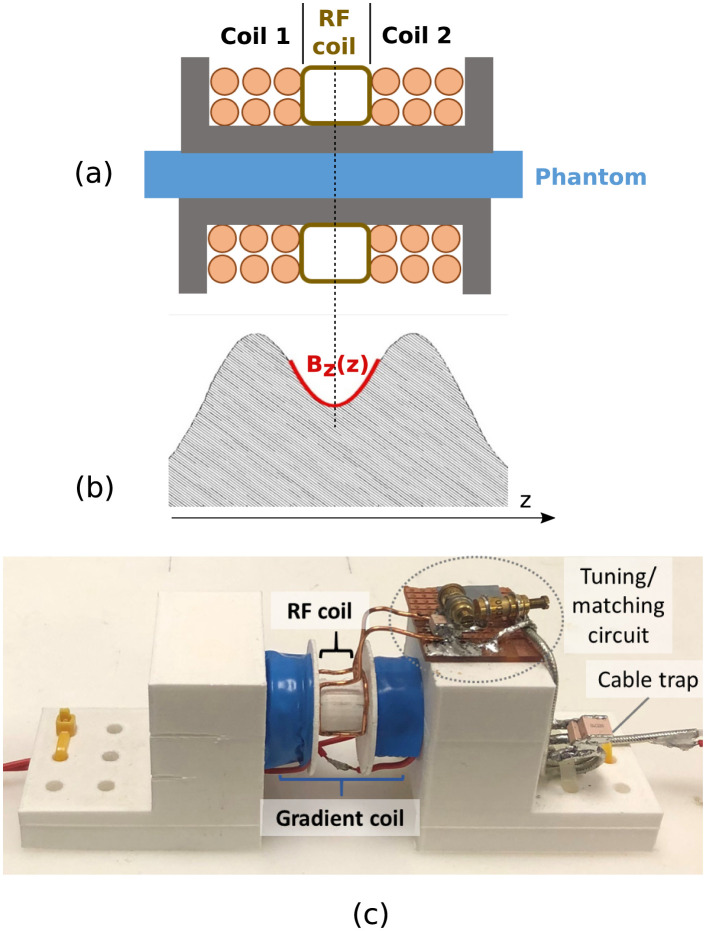
Coil insert. (a) The schematic of the prototype coil insert: The Helmholtz pair consists of two individual coils with a length of 11 mm and 27 windings of 28 AWG insulated copper wire each. The inner diameter of the coil is 10.5mm, the outer diameter 12.5mm. The separation between coil 1 and coil 2 is 12mm. A RF saddle coil (transmit and receive) is fitted into the separation between the Helmholtz pair. The coil insert is unshielded. (b) Gradient field B_*z*(*z*)_ generated by the Helmholtz coil along the z-axis. Due to Maxwell’s equations the minimum along z is in reality a saddle point. (c) Picture of the coil insert: a 3D printed structure holds the gradient coil (wrapped in blue tape). The RF saddle coil is fitted between the Helmholz pair. The tuning/matching circuit for the RF coil is mounted on the structure as well as a cable trap (to suppress common mode currents).

The gradient coil is the core element of the experimental set-up and is capable of generating strong Z2-shaped magnetic gradient fields. The desired shape and duration of the gradient pulse is controlled with an arbitrary waveform generator (AWG) whose output voltage is fed to a low-cost open source current driver [25]. The voltage is translated into a current which is subsequently supplied to the gradient coil via a feedtrough filter. The gradient coil consists of a Helmholtz pair which is modified in the sense that the separation between the individual coils is larger than their radius. The design details of the coil are summarised in the schematic of the coil insert shown in Fig 7.

Simulations of the magnetic field inside the gradient coil using the open-source software FEMM (finite element methods magnetics) [26] with the specifications of the gradient coil yield a curvature of *G*_2_ = 16 T m^-2^ around the coil’s centre for a coil current of 1 A. Additionally, the field inside the gradient coil was measured using a Gauss meter to confirm the field has the desired shape. More information can be found in S1 Fig. Limited by the dimensions of the gradient coil, only a small sample fits inside the bore of the insert. Also, signal away from the centre of the gradient field (where the saddle point is) is expected to be attenuated by the increasingly linear behaviour of the gradient field. This means only a small volume of the sample yields signal in the diffusion experiments and hence it is important for the RF coil to be positioned as close as possible to the sample. A small RF saddle coil [27] was built that fits between in the separation of the Helmholtz pair. Tuning the coil so that the resonance frequency of the electrical coil circuit matches the resonance frequency of nuclear MR of water at 3T (127 MHz) and matching the coil impedance was achieved with two capacitors (one in series and one in parallel to the RF coil).

### Anisotropic fibre phantom

For this paper, a phantom made of synthetic Dyneema^®^ fibres is chosen. The design and manufacturing of the phantom is based on the work published by Fieremans et al. [20, 21] which was found to mimic the diffusion behaviour of brain WM. Dyneema^®^ is particularly well suited since its magnetic susceptibility is close to water and the individual fibres are, with a diameter of 20 μm, on the right scale to impede and hinder the diffusion of water molecules. They are made of ultra-high-molecular-weight polyethylene, a hydrophobic material.

A phantom was manufactured by tightly packing the fibres and holding them together with the help of low-temperature shrink tube (poly-olefin). The fibre bundle was placed inside the shrink tube under water and the water temperature was increased to 90°C to ensure the shrink tube sits as tight as possible around the fibre bundle. This is paramount to achieve high anisotropy, it also traps water molecules between the individual fibre strands which give rise to the MR signal later in the experiments. Repeated squeezing removed remaining air bubbles in the phantom. The phantom was then placed in a NMR tube (diameter 10 mm) filled with fomblin to avoid phantom-air interfaces without adding MR visible signal.

### Experiments

#### Methods

All experiments were performed on a 3T Achieva Philips Scanner. First, the FA of the fibre phantom was determined in a six-direction DTI experiment, using the scanner’s linear gradient with the b-values = [0, 2000].

**Fig 8:**
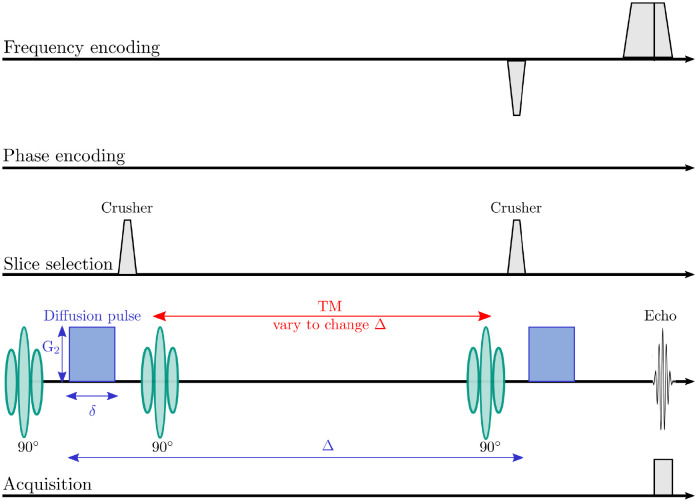
The 1DFT STEAM sequence: The first 90°excitation pulse is followed by the first diffusion gradient pulse. A second 90°pulse rotates the net magnetisation back into the longitudinal plane; there it is stored and the net phase preserved. The spins are allowed to mix before a third 90°pulse is applied, followed by the second diffusion gradient. Δ is varied by modifying the mixing time TM between the second and third 90°pulse.

Then, the dMRI experiments with quadratic gradients were performed. For this, a 1DFT stimulated echo acquisition mode (STEAM) sequence is chosen that enables the use of long diffusion times since T2 signal decay is uncoupled from the diffusion time [28]. As shown in the sequence diagram in Fig 8, the 1DFT sequence has no phase encoding gradients, i.e. spatial resolution of the measured MR signal is only obtained along the z-axis. This allows the acquisition of repeated phase measurements over time in one experiment. During each signal readout, a line is obtained that represents the projection of the MR signal on the z-axis at this point in time. Different lines contain information about the MR signal at a different time, but at the same spatial location.

In the experiments, alternating lines with and without diffusion-gradients were acquired. The non-weighted data serves as baseline data to correct for phase changes that are induced by other than the diffusion gradients, for example, by the spatial encoding gradients of the sequence. With the TTL (transistor-transistor logic) signal of the MR scanner, the AWG is triggered to generate a diffusion pulse. To avoid problems with eddy currents due to sharp rising times, half the period of a sine wave is chosen as pulse shape. Data was acquired for two different Δ (Δ_1_ = 243ms, Δ_2_ = 443ms). These were realised by changing the mixing time TM as shown in Fig 8, while the pulse duration *δ* = 30 ms, the repetition time TR = 3000 ms and the echo time TE = 90 ms were unchanged. For each Δ, 512 lines with and 512 lines without diffusion weighting were acquired. The curvature of the gradient field was *G*_2_ = 9.6 T m^-2^ (which corresponds to a coil current of 600 mA).

The dMRI experiments with quadratic gradients were performed first on the isotropic sample a NMR tube filled with agar gel and then on the anisotropic fibre phantom. The experiment in the fibre phantom was repeated. To calculate the mean of the measured phase, all pixels with signal were considered. The mean phase of the baseline data was subtracted from the mean phase of the diffusion weighted data to obtain a mean phase that stems from the diffusion process. The error in the mean of the measured phase was obtained by error propagation of the standard errors of the baseline and diffusion weighted phase values. This was done for each experiment and each Δ. The expected phase change according to the analytic model was also determined. For this, cylinder symmetry was assumed and the ratio of transversal to longitudinal diffusion coefficient *D*_⊥_/*D*_∥_ was based on the FA value found in the DTI experiment. The values for the gyromagnetic ratio *γ* and the longitudinal diffusion coefficient for water at a temperature of (*D*_∥_ = 2.0×10^−9^ m^2^ s^-1^, 20°C) were based on literature values [19].

#### Results

**Fig 9:**
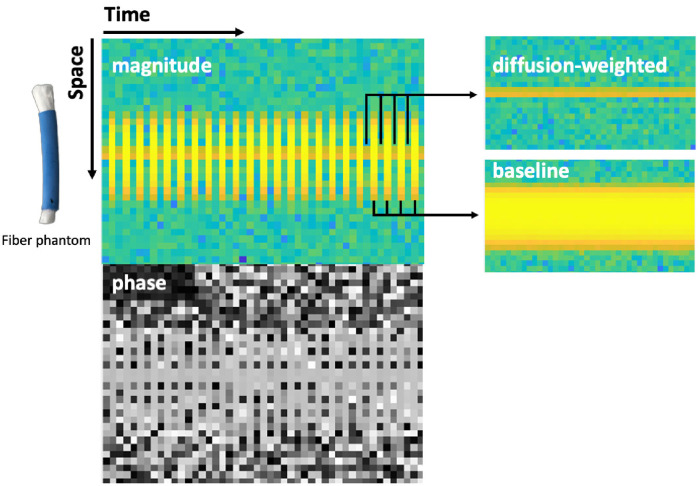
The magnitude and phase image acquired with the 1DFT STEAM sequence (left). The space dimension of the images correspond to the z-axis of the coil insert as shown in Fig 7.Lines with and without diffusion-weighting are alternated. On the right, the separated magnitude images with diffusion weighting (top) and without (bottom) are shown. When the diffusion gradients are on, signal away from the saddle point is dephased.

The DTI experiment yielded an anisotropy FA = 0.5. The magnitude and phase images of one of experiments with the fibre phantom can be found in Fig 9. On the top left, the magnitude image shows a stripe-like pattern caused by the on and off switching of the diffusion gradients. On the right, the magnitude image is split into two, showing the data with diffusion weighting and the baseline signal (no diffusion-weighting) separately.

**Fig 10:**
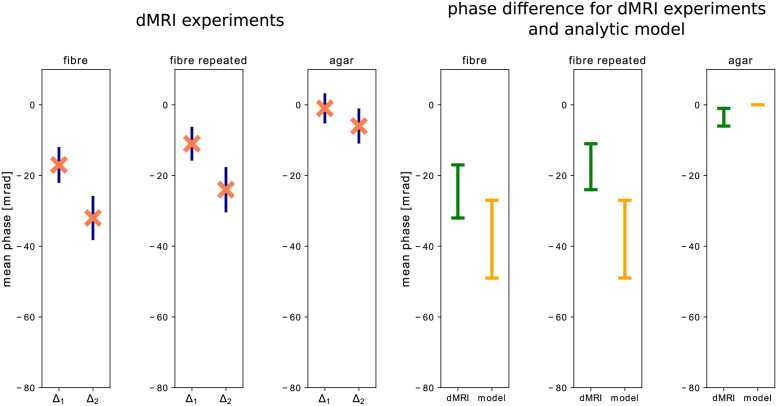
The results of the dMRI experiments: The three plots on the left show the mean phase over time for the pixel associated with the saddle point of the gradient field for Δ_1_ and Δ_2_. The acquired baseline was used to correct for phase errors. The resulting error was determined using error propagation. The three plots on the right compare the difference of the measured mean phase (green) between Δ_1_ and Δ_2_ to the difference estimated by the analytic model (orange). The upper and the lower bar mark the phase for Δ_1_ and Δ_2_ respectively.

To calculate the mean of the measured phase, all pixels with signal were considered. In case of diffusion weighted images, only one spatial location yields signal which totals to 1×512 data points (space×time). For the baseline images, 8×512 pixels were used. The error bars for two identical experiments with the fibre phantom and for the experiment with agar are shown in the left half of Fig 10. The right half shows the difference of the mean phase between Δ_1_ and Δ_2_ for the dMRI experiments and the analytic model. Table 5 gives the difference in phase change between the two Δ.

**Table 5:** The difference in the mean phase between the two separation times Δ_1_ and Δ_2_. The error was computed by error propagation of the errors presented in Fig 10.

Phantom	*φ*_2_ − *φ*_1_	Error	Unit
Agar dMRI	-5	6.5	mrad
Fibre dMRI	-15	6.2	mrad
Fibre dMRI repeated	-13	7.9	mrad
Fibre analytic model	-22		mrad

Comparing the diffusion weighted and baseline images, two things can be observed: even when strong quadratic gradients are applied, large portions of the signal are preserved. Moreover, when comparing the spatial extent of the signal it becomes clear that with diffusion weighting, only signal from a small region, which corresponds to the centre of the gradient field, survives. This also justifies the use of a 1DFT sequence with spatial encoding only along one direction. In both of the experiments with the fibre phantom, the mean phase change is larger for longer Δ (and hence more diffusion weighting). The results show that increasing Δ by approximately a factor of two leads to approximately a double change in the mean of the measured phase. In the isotropic agar phantom, approximately zero change in phase was observed between the two separation times. All three experiments show smaller standard error for shorter Δ, since more signal is available.

## Discussion

We have introduced a novel approach to diffusion-weighted MRI that uses non-linear diffusion gradient fields. In doing so, diffusion is encoded in the signal phase, opposed to classical dMRI, where diffusion encoding is achieved by an attenuation of the signal magnitude. The theoretical background was given and based on this, an analytic model for the net phase was derived. The model predicted that diffusion in anisotropic media leads to a phase change that is dependent on both properties of the underlying media and experiment specific parameters, such as the separation time between diffusion gradients or their duration. No change in net phase is expected for isotropic diffusion. To study more complex structures that go beyond the analytic model, a Monte Carlo simulation framework was described. Existing Monte Carlo simulation tools for Brownian motion were used to generate trajectories for a large number of diffusing spins. The net phase is obtained as the ensemble average of the phase accumulations of all individual spins. First, the simulation framework was used to validate the analytic model against simulations that reflect the assumptions made for the analytic model. The simulated values for the net phase are in excellent agreement with those predicted by the analytic model. The results confirmed that there is no phase change for isotropic diffusion and that, in the anisotropic case, the net phase depends on the degree of anisotropy. Moreover, for an anisotropic medium with constant degree of anisotropy, the net phase was also found to depend on the diffusion weighting. Similar findings were reported for simulations with a numerical fibre phantom made of parallel cylinders. The simulations in the fibre substrates were performed with both quadratic and linear diffusion gradients yielding comparable results for the FA.

To gain further insight, in a realistic setting that goes beyond the assumptions made in the analytic model, the proposed approach was tested in dMRI experiments with a prototype coil insert consisting of a small-scale gradient and RF coil. It could be observed that signal in diffusion-weighted data is spatially concentrated in a small region around the centre of the gradient field which coincides with its saddle point. Away from the saddle point, the increasingly linear behaviour of the gradient field dephased the signal. In qualitative agreement with the analytic model, an increase of the diffusion weighting by approximately a factor of two lead to an increase in the mean of the measured phase by approximately a factor of two in the anisotropic fibre phantom. Comparing the results from the two dMRI experiments within the anisotropic fibre phantom showed that the repeated experiment agreed less well with the analytic model. Possible reasons are hardware instabilities or higher order gradient non-linearities contributing to a systematic error in the phase measurements that need to be addressed in future work.

Due to the novel, introductory nature of this work, there are still some limitations. Both the validation simulation and the simulation in the fibre phantom were too simple to be representative of human tissue. The validation simulation was set up to reflect the idealised assumptions made to derive the analytic model. This means that the diffusivity was homogeneous along each direction and did not have any local variation. Although this was partly addressed by adding solid cylinders as obstacles in the fibre simulations, this work could benefit from the recent advances made in the development of realistic numerical diffusion MRI phantoms, for example using contextual fibre growth to mimic fibre genesis. [29]. The simulations, both for quadratic and linear gradients, only considered effects that diffusion and diffusion gradients have on the MR signal (magnitude and phase). Signal decay due to relaxation effects, noise or scanner imperfections were neglected.

While the initial experimental results are very promising and support the introduced theoretical framework, there is further work required. Thus far, only the dependence of the signal phase on different diffusion-weighting was studied with two mixing times. Future experiments can be extended to consider more mixing times and the influence of other scan parameters (pulse duration and gradient strength). The experiments were designed to maximise the change in phase to be able to measure it with the present noise and phase errors. Hence the large number of data points acquired which naturally meant that the scans were very time consuming. Therefore, improving the hardware is an important part of future work. The prototype coil insert in its current form is very simple. It does not have any shielding, which could be a reason for the observed phase errors in the measurements. The performance of the coil insert would potentially benefit from shielding and better filtering of the input signal. Also, replacing the current driver with a low noise gradient amplifier could reduce noise and phase errors. Moreover, an improved design for the receiver coil could help to be more sensitive to the signal and thus improve SNR.

The proposed method also needs to be evaluated on more samples, such as fibre phantoms which vary in anisotropy, and also on realistic samples, e.g. ex-vivo tissue samples or small animals. The current coil is too small for the latter. With the current hardware, the close-fitting coils are necessary to generate the required gradient strength. A bigger coil would require stronger currents and therefore more work on the hardware, also including safety aspects.

Since the proposed method represents a novel way of encoding diffusion in the phase, the obtained images are not directly comparable to images obtained with conventional dMRI. Hence, a comparison between biomarkers derived from the signal magnitude of dMRI with linear gradients and the same biomarkers derived from the signal phase of dMRI with quadratic gradients would be required in future work as further validation of the method.

The experimental set up in its current form provides only single-point measurements, since signal away from the saddle point of the Z2 gradient field is increasingly dephased by the linear components of the field. To address this limitation, future work could include superimposing additional linear gradient fields to change the position of the saddle point. A further consequence of this dephasing away from the saddle point is that an increasing the gradient strength decreases the effective sensitive volume that contributes to the signal. This means that the information in the phase stems from a more localised region, but it can also affect the SNR. To what extent this is the case has to be investigated in future experiments.

An interesting direction for future work could be the combination of the proposed Z2 shaped field with other non-linear fields. Using the proposed Z2 shaped gradient field yields a signal phase that contains information about the diffusion process in 3D. Combining phase measurements with this field with those from other second order non-linear magnetic fields (e.g. XY, X2 -Y2) could offer the potential to gain additional information about the spatial differences in the diffusion process. Moreover, the fibre phantom was positioned inside the gradient coil such that its main direction of anisotropy aligns with the dominant component of the gradient field. This was chosen to maximise the expected phase change but is not necessarily a realistic scenario. Any alignment that is not parallel to the z-axis reduces the measured phase. A more sophisticated gradient coil with additional gradient non-linear gradient fields as just mentioned, or with the option to rotate the gradient field, would enable measurements along different directions. Analogous with standard dMRI, the quadratic diffusion weighting presented here could be combined with recent advances in standard dMRI, including the use of more complicated sequences such as double diffusion encoding (DDE) [30, 31], oscillating gradient spin-echo OGSE [32], etc.) and complex modelling to further quantify microstructure in different anatomies. dMRI using non-linear gradient fields could offer improved sensitivity to microstructure and provide valuable information that is currently inaccessible. One potential application is anomalous diffusion, which has been previously proposed to be a better biomarker for diffusion in complex media [33, 34]. In [35], Hall and Barrick introduced an alternative signal model based on anomalous diffusion which can be used to extract information about the fractality of the underlying tissue. However, the nature of the model means that it struggles with low SNR. Hence, a method that preserves the signal magnitude, such as the method proposed in this work, could increase sensitivity and facilitate the extraction of such a biomarker. Though, the question of whether this potential SNR benefit can be realised would have to be investigated in future research. In future work, this method and model could be extended to account for anomalous diffusion, which would allow to probe the complexity of tissue. The same simulation framework used in this paper could be used to study the effect of diffusion of structures with known fractality on the net phase.

## Conclusion

Diffusion MRI traditionally uses linear gradients to encode diffusion in the attenuation of signal magnitude This paper introduces a novel approach to dMRI that uses a quadratic Z2 diffusion gradient field. This allows the encoding of anisotropic diffusion in the phase of the MR signal whilst preserving the signal magnitude around the saddle point of the gradient field. Diffusion and phase evolution of a spin ensemble in the presence of such a gradient field was studied in theory, simulation and dMRI experiments with an anisotropic phantom made of synthetic fibres. Initial experimental results, though still affected by hardware imperfections and measurement errors, support the phase behaviour expected from theory and simulations.

## Supporting information

S1 File Detailed derivation of the analytic model. (PDF)

S1 Fig Simulation and measurement of the magnetic field of the gradient coil. (a) The magnetic field generated by the prototype gradient coil was simulated using the open-source software FEMM. The plot shows the magnitude of the B-field along the z-direction and along the x/y-axis. (b) The magnitude of the magnetic field was also measured using a hand-held Gauss meter. The plot shows the measurements along the z-directions.
(EPS)
