# Supplementary material for: Diffusion phase-imaging in anisotropic media using non-linear gradients for diffusion encoding
Source: PLoS One. 2023 Mar 30;18(3):e0281332. doi: 10.1371/journal.pone.0281332 (PMC10062566; doi:10.1371/journal.pone.0281332)
Supplement: S1 File — (PDF) [file pone.0281332.s001.pdf]

Supporting information:

S1 - Derivation of the analytical model

In the following, a more detailed derivation of the analytic model is given. Starting from Eq. 7 we obtain

$$\begin{aligned}\varphi_2 &= \gamma \int_0^T dt \int_{-\frac{\Delta z}{2}}^{+\frac{\Delta z}{2}} dz\ G_2 z^2 \frac{1}{\sqrt{4\pi D t}} \exp\left(-\frac{z^2}{4Dt}\right) \\ &= \gamma G_2 \int_0^T dt \int_{-\frac{\Delta z}{2}}^{+\frac{\Delta z}{2}} dz\ \frac{\sqrt{4Dt}}{\sqrt{\pi}} \left(\frac{z}{\sqrt{4Dt}}\right)^2 \exp\left(-\left(\frac{z}{\sqrt{4Dt}}\right)^2\right)\end{aligned} \tag{1}$$

Substitution ($u := \frac{z}{\sqrt{4Dt}}$) and integration yield

$$\varphi_2 = \gamma G_2 \int_0^T dt \left[\underbrace{-\sqrt{\frac{Dt}{\pi}} z\ \exp\left(-\frac{z^2}{4Dt}\right)}_{\text{yields 0}} + Dt\ \mathrm{erf}\left(\frac{z}{\sqrt{4Dt}}\right)\right]_{-\frac{\Delta z}{2}}^{+\frac{\Delta z}{2}} \tag{2}$$

The left term yields zero. The Gaussian error erf(x) is defined by

$$\mathrm{erf(x)} \ = \frac{1}{\sqrt{\pi}} \int_{-x}^{+x} dt\ \exp\left(-t^2\right) \tag{3}$$

Moreover, $\Delta z = 1$ mm, D = 2.3·10^{-9} $\left[\frac{m^2}{s}\right]$ and $T = 250$ ms were assumed. The choice of z, D and T made for solving the spatial integral does not limit the generality of the solution, since combinations of values within the range encountered in typical diffusion experiments yield ±1 for the term erf(.).

This leaves

$$\varphi_2 = \gamma G_2 \int_0^T dt\ 2Dt \tag{4}$$
